# Structure of Leaf Galls in *Clusia fluminensis* Planch and Triana (Clusiaceae): Sex-Biased Development in a Dioecious Host Plant

**DOI:** 10.3390/plants10010020

**Published:** 2020-12-24

**Authors:** André Guimarães, Ricardo Vieira, Ana Vieira

**Affiliations:** 1 Laboratório de Farmacobotânica, Faculdade de Farmácia, Universidade Federal do Rio de Janeiro, CCS, Avenida Carlos Chagas Filho 373, Cidade Universitária, Ilha do Fundão, Rio de Janeiro, RJ 21941-902, Brazil; acmvieira@pharma.ufrj.br; 2 Laboratório de Morfologia Vegetal, Departamento de Botânica, Instituto de Biologia, Universidade Federal do Rio de Janeiro, CCS, Avenida Carlos Chagas Filho 373, Cidade Universitária, Ilha do Fundão, Rio de Janeiro, RJ 21941-902, Brazil; vieirarc@biologia.ufrj.br

**Keywords:** Cecidomyiidae, gall, *Parazalepidota clusiae*, sex-biased metabolism, sex-biased interactions

## Abstract

Galls are remarkable parasite–plant interactions that develop in different organs. They are induced by various organisms which manipulate or reprogram plant development. Galls in dioecious species and their effects on the host plant are seldom described in the literature. This paper presents a novel study of galls in a dioecious plant of the neotropical region. Our study aimed to analyze gall development and describe morphological and metabolic changes in galled leaves caused by Cecidomyiidae (Diptera) in *Clusia fluminensis* (Clusiaceae), a dioecious plant from Brazilian *restinga*. We investigated male and female individuals to detect different responses and sex-biased interactions. The non-galled leaves of female and male individuals of *C. fluminensis* exhibited similar anatomical structures. Nevertheless, galls developed only in male individuals. The activity of the Cecidomyiidae led to several morphological and anatomical changes, such as the hypertrophy of the leaf blade, especially the chlorenchyma. Our results indicated an interesting exception for the pattern of storage of lipids and starch in Cecidomyiidae galls, and sex-biased development in a dioecious plant, with the variation of metabolic compounds, especially phenolics and flavonoids, which may inhibit gall development in female individuals.

## 1. Introduction

Clusiaceae species are widely distributed in the pantropical region, with several species found in different Brazilian ecosystems, notably in “restinga” habitats. The “restinga” habitats are alongside the Brazilian coast, distinguished by sandy coastal plains, with open vegetation of shrubs and low forests further inland [1,2]. In these habitats, several dominating species may be characterized by the abundance of individuals or the plant cover of a few taxa, such as *Clusia* [3,4,5]. Species of *Clusia* are typical of the neotropical region with 250–300 species [6,7]. Several species play an ecological role as nurse plants [8,9]. *Clusia fluminensis* Planch and Triana (Clusiaceae) is an obligate CAM (crassulacean acid metabolism) species [10] and some of the most abundant species in the *restingas* of Rio de Janeiro. This species exhibits dioecism, with male and female individuals in the shrub thickets of *restingas* [3].

Among the diverse interactions of *C. fluminensis* with other organisms is the formation of galls in leaves which are induced by *Parazalepidota clusiae* Maia, 2001, a species of Cecidomyiidae (Diptera) [11]. Galls represent a unique type of ecological interaction between inducer organisms and plants and are defined as anomalies in the plant development of parasitic origin that affect cellular differentiation or the growth of plant structures and organs [12]. Plant galls can be viewed as novel plant organs whose development is triggered by the induction of existing plant developmental pathways in unusual (ectopic) places and combinations [13]. Different organs, both vegetative and reproductive, may be altered by the inducer organism [14,15,16,17]. The development of galls on plants occurs in diverse structural patterns directly influenced by the inducer’s activity. According to Sanver and Hawkins [18], gall-inducers are considered microhabitats engineers since they provide nutritional resources and habitat to be explored.

Additionally, these inducers’ construction of shelter increases larval survivorship and reflects remarkable interactions between insects and plants [19]. Some studies indicated that galls become sinks of nutrients, compete for nutrients, and redirect resources from other organs [20]. Their effects can also be detected in the galled organs and the surrounding parts due to the drainage of nutrients to those galled organs [17,20,21]. Thus, the adjacent leaves may exhibit negative impacts of the development of galls reflected in morphological, anatomical, and physiological traits [22,23]. Furthermore, other studies analyzed the differences in the intensity of herbivory and the development of leaf galls related to the sex of the host plants. They indicated that male individuals were more infested than the female ones [24,25,26]. Hartley [27] revealed changes in the synthesis of nutrients and chemical compounds between galled and non-galled tissues.

Clusiaceae species have been described in the last decades regarding their chemical composition, especially the secondary metabolites, such as xanthones, triterpenoids, flavonoids, lactones, organic acids, and several oxidized and poly-isoprenylated benzophenones (PBDs). Extracts from the fruits and flowers of *C. fluminensis* with clusianone were effective against *Aedes aegypti* Linnaeus, 1762 development, and showed potential as a biopesticide [28,29,30,31,32,33]. Another study analyzed the effects of leaf galls in the morphology and chemical composition in female and male individuals of *Clusia lanceolata* Cambess. [14]. Nevertheless, changes in leaf development and secondary metabolites of organs with galls in *Clusia* and other taxa from these harsh environments such as “restinga” remain unknown. An intriguing question refers to morphological, developmental, and chemical features of gall development. Are they solely related to the influence of the gall inducer on development pathways or to possible variation regarding sex in dioecious host plants as well? We hypothesize that such changes may reflect the activity of the inducer and sex-biased metabolic responses.

Therefore, to bridge this gap, this paper seeks to: (i) study the alterations in the morphology and anatomy of galled and adjacent leaves in both sexes and (ii) investigate the histochemical gradient and chemical composition of galled and adjacent organs in both sexes of *C. fluminensis.*

## 2. Results

### 2.1. Morphological Characterization

**Figure 1:**
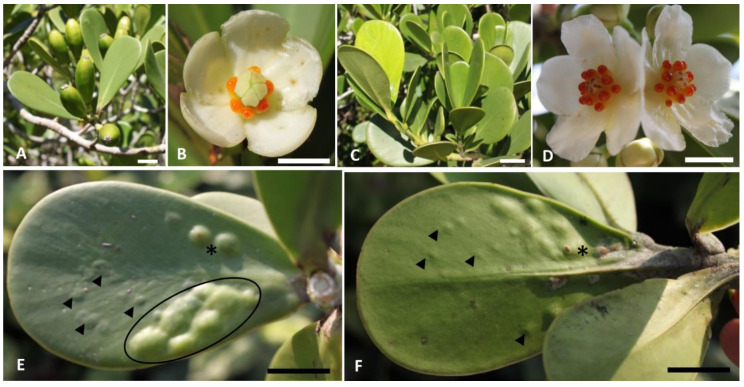
*C. fluminensis.* Female (**A**,**B**) and male individuals (**C**–**F**) with leaf galls induced by *Parazalepidota clusiae* (Cecidomyiidae) (**E**,**F**). (**A**) Non-galled leaves and fruits in the initial stage of development. (**B**) Female flower. (**C**) Non-galled leaves of a male individual. (**D**) Male flowers. Note the resiniferous stamens. (**E**) Adaxial surface of the galled leaf in different stages of development. Note the beginning of surface hypertrophy in initial stages (arrowhead), mature adjacent galls forming a continuous hypertrophied gall system (circle), and pre-senescent isolated galls (*). (**F**) Abaxial surface of the galled leaf. Note the beginning of surface hypertrophy in initial stages (arrowhead) and development of the exit channel in pre-senescent galls (*). Bars: (**A**–**F**) = 1 cm.

The non-galled leaves of female and male individuals are latescent, obovate, glabrous, coriaceous, with short and thick petiole, rounded apex, attenuate base, and entire margin. They are bright green colored in both faces (Figure 1A–D). The occurrence of galls was observed across the leaf blade with progressive hypertrophy of both surfaces from March to June. We did not observe the development of galls in female individuals.

In the initial stage of the development of galls, we observed the beginning of the hypertrophy of the leaf blade in both faces, which continues throughout development, and galls of the same growth season (March–June) in different stages developing in the same galled leaf (Figure 1E,F). Galls exhibited a lenticular shape with no changes in color, except in the senescent stage, in which dark green galls with exit channels of the inducers were evident in both faces (Figure 1E,F). The senescent galls became isolated from the remaining functional leaf area.

The number of galls in each leaf was variable and they were often observed in leaves from the first to the third nodes from the previous vegetative branching during the rainy season (ending in March). We observed 1–18 galls distributed throughout the leaf blade, with no preferential pattern regarding the leaf surface. The average diameter of isolated galls ranged from 0.2 ± 0.1 cm in the initial stages and 0.49 ± 0.1 cm in the mature and senescent development stages. The galls developed isolated, or they sometimes fused, forming an integrated mass of new tissues (Figure 1E,F). However, the larval chambers remained isolated. The Cecidomyiidae larva developed in individual larval chambers with only one inducer.

### 2.2. Anatomical Characterization

#### 2.2.1. Non-Galled Leaves

**Figure 2:**
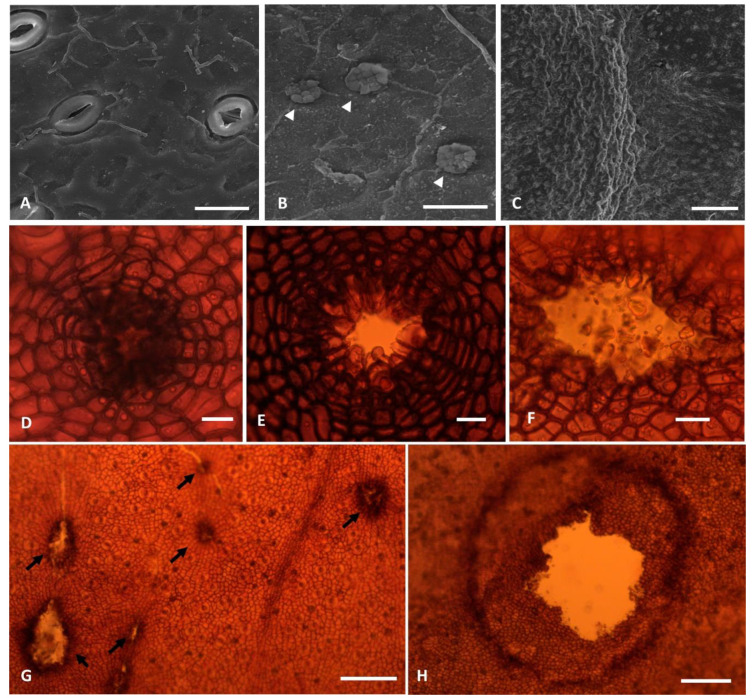
*C. fluminensis.* Epidermis of non-galled leaves (**A**,**B**) and galled leaves (**C**–**H**) of male individuals. (**A**–**C**) Scanning electron microscopy. (**A**) Abaxial surface showing stomata and straight cuticle with smooth to finely warty layers. (**B**) Cork warts (arrowhead) from the adaxial surface. (**C**) Abaxial surface of the gall region. Note the rough surface, with thick crusts, due to mesophyll hypertrophy. Stomata were abundant next to galls. (**D**–**F**) Different stages of development of the cork-warts. (**G**) Leaf epidermis of galls. Note the cork-warts in each gall. (**H**) Exit channel. Note the degenerating cells forming a circle around it, which isolates the degeneration of epidermis from the remaining cells. Bars: (**A**,**B**) = 20 µm; (**C**) = 200 µm; (**D**–**F**) = 20 µm; (**G**) = 200 µm; (**H**) = 100 µm.

**Figure 3:**
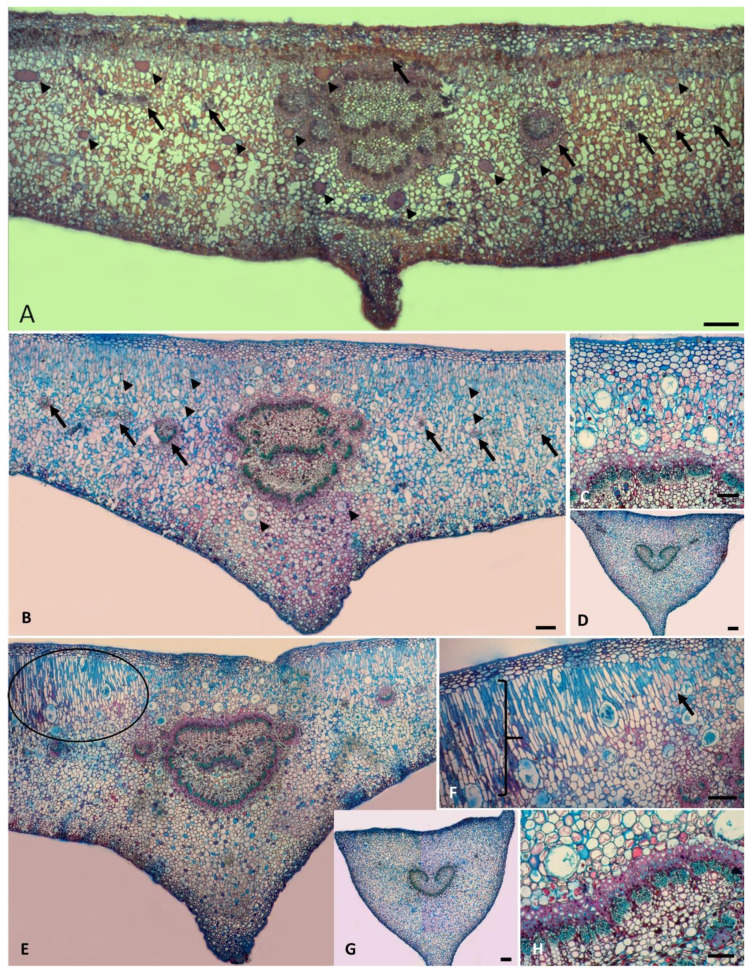
*C. fluminensis.* Non-galled leaves of female (**A**) and male individuals (**B**–**H**). (**A**) Transverse section (TS) of non-galled leaf in the midrib region. Note the flat-convex shape with a wing projection in the abaxial surface, several laticifers across the mesophyll (arrowhead), and the continuous palisade parenchyma (arrow). (**B**,**C**) Non-galled leaves of a male individual. Note the gender differences, regarding the flat-convex shape, and the abundance of laticifers surrounding the vascular region and across the palisade. (**D**) TS of non-galled petiole with several laticifers and idioblasts with phenolics across the cortex. Note the vascular system in open arc and the margins flexed downwards. (**E**) TS of the galled leaf, showing hypertrophy of palisade parenchyma and abundance of laticifers towards gall tissues (circle). (**F**) Adaxial surface of the galled leaf with galls Note the beginning of surface hypertrophy (arrow) from divisions and elongation of palisade cells (}) towards the gall system. (**G**) TS of petiole of galled leaf showing hypertrophy of cortex and abundant idioblasts with drusa. (**H**) Details of the midrib of galled leaf. Note the hypertrophy of vascular tissues and perivascular sclerenchyma sheath. Bars: (**A**,**B**,**D**) = 200 µm; (**C**,**E**,**F**) = 100 µm; (**D**,**G**) = 500 µm.

The non-galled leaves were hypostomatic with dorsiventral mesophyll and similar characteristics in both sexes. The surface exhibited a straight cuticle with smooth to finely warty layers (Figure 2A,B). The adaxial surface of leaves of male individuals showed oblong to elliptical ordinary cells of various sizes. The anticlinal walls were thick and straight. The abaxial surface exhibited similarly shaped cells but smaller with thicker walls (Figure 3A–C). The stomata were paracytic, elliptical with kidney-shaped guard cells, and scattered throughout the epidermis (Figure 2A). We also observed cork-warts in both faces, especially in the abaxial surface (Figure 2B).

In the intercostal region, the leaf blade revealed uniseriate epidermis with conspicuous cells and thicker cuticle in the adaxial face, multiseriate hypodermis with 2–4 layers of elliptical cells in leaves of male individuals and 4–7 layers in female ones (Figure 3A). Idioblasts with drusa were abundant in both sexes (Figure 3A,B). We also observed 3–5 layers of palisade parenchyma in male and 2–3 layers in female individuals and 15 layers of spongy parenchyma in both sexes. Resin ducts were abundant, especially in the adaxial portion. However, there were fewer in leaves of female individuals (Figure 3A,B). Several vascular collateral bundles were found in the median part of the mesophyll. The midrib region revealed similar epidermis and hypodermis, 3–5 layers of collenchyma in the abaxial face, 15 layers of ground parenchyma, and the palisade parenchyma continuum in female individuals. The vascular system consisted of 30 external bundles arranged in a closed arc and 2–5 inner bundles. We also observed a sheath of sclerenchyma cells and several resin ducts around the vascular system. The female individuals exhibited leaves with a straight projection in the abaxial face, whereas the male individuals had leaves with a flat-convex shape (Figure 3A,B).

The distal portion of the petiole of non-galled leaves exhibited a flat-convex shape, with two wings on the adaxial surface and one on the abaxial one, uniseriate epidermis with a thick cuticle, three to four layers of collenchyma, and several idioblasts with drusa in the sub-epidermal layers, 10–15 layers of ground parenchyma cells, abundant secretory ducts, and idioblasts with phenolics in the cortex. The vascular system consisted of 25–30 collateral bundles arranged in an open arc, and the margins flexed downwards (Figure 3D).

#### 2.2.2. Galled Leaves

The epidermis of galled leaves was similar to the non-galled ones, with thicker anticlinal walls of ordinary cells on both surfaces (Figure 3D). The surface of galls became rough, with thick crusts in both faces. Stomata were abundant next to galls (Figure 2C). Hyphae were often present on both surfaces, especially next to the exit channel in the senescent stage. The cork-warts exhibited the beginning of wall thickening and suberin deposition since the initial stages of gall development (Figure 2D–F). These structures developed from divisions of ground cells or stomata (Figure 2G,H). In the mature stage of development, they exhibited concentric arrangement of peripheral cells (Figure 2E) and a central cavity (Figure 2D–H), which were revested by suberin in some cases (Figure 2G). The cork-warts were abundant and variable in size in the senescent stage, especially on the abaxial surface.

The midrib region adjacent to the larval chamber showed a slight flattening of the hypodermis and hypertrophy of the palisade parenchyma by periclinal divisions and elongation of the cells. Chloroplasts were continuously present only in the outer 2–3 layers, and cells from median hypertrophied palisade parenchyma were highly vacuolized and rich in lipids towards the larval chamber. Spongy parenchyma and intercellular spaces were often preserved. Resin ducts became more abundant around the vascular system and throughout the mesophyll towards the larval chambers. We also observed the development of collenchyma, notably in the abaxial face, the thickening of the perivascular sheath, and more idioblasts with drusa (Figure 3E,F,H).

The petioles of galled leaves revealed similar patterns compared to those without galls. We highlight the hypertrophy of the cortical region, with the increase of layers of the ground parenchyma, the development of collenchyma throughout the sub-epidermal portion, especially in the adaxial surface, and abundant idioblasts with drusa (Figure 3G).

**Figure 4:**
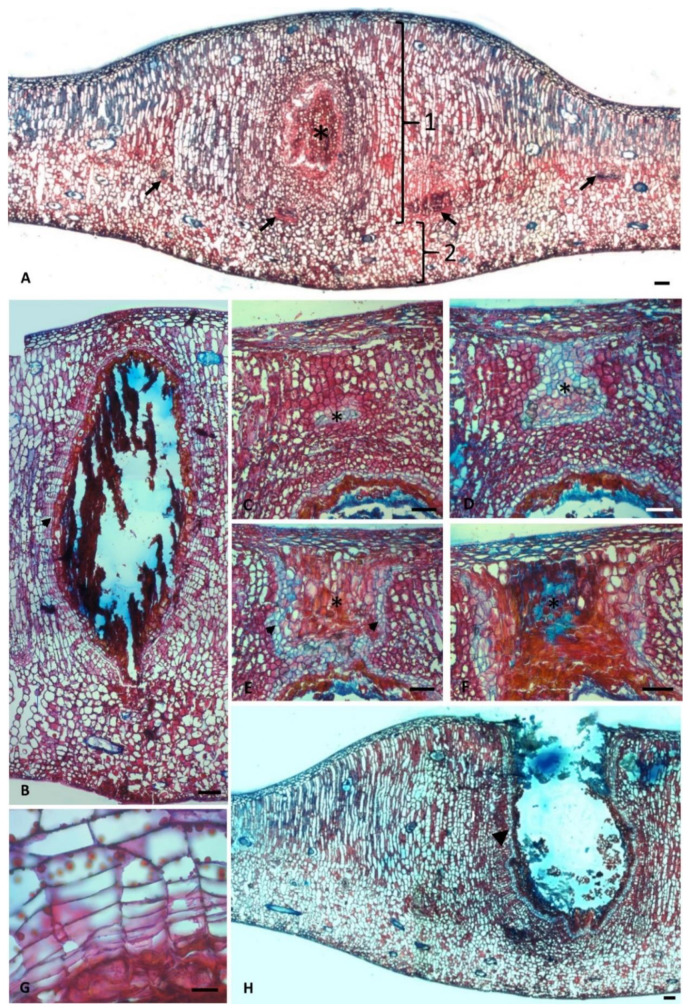
*C. fluminensis.* TS of galled leaves of male individuals. (**A**) Note the hypertrophy of the mesophyll, due to mainly the increase of palisade layers (1), whereas the spongy parenchyma (2) remains like the non-galled regions, but with flattened cells; several collateral bundles across the median region of the mesophyll (arrow), and the nutritive tissue from the larval chamber (*). (**B**) Larval chamber of mature gall. Note the organization of meristematic cells (arrow), continuously forming the nutritive tissue all around the larval chamber. (**C**–**F**) Progressive steps of the formation of the exit channel, through degeneration of parenchyma cells (*) between larval chamber and epidermis. Note the development of meristematic cells (arrow) stablished from the epidermis to the nutritive tissue, isolating the senescent tissues from the remaining tissues. (**G**) Details of the meristematic cells developed from the surrounding parenchyma cells that continuously form the nutritive tissue. (**H**) Senescent gall. Note the exit channel with degenerating cells and the meristem layers that isolate the exit channel from the remaining tissues. Bars: (**A**) = 200 µm; (**B**–**F**,**H**) = 100 µm; (**G**) = 20 µm.

The leaf blade adjacent to galls in the initial stage of development exhibited a flattening of both epidermis and hypodermis, hypertrophy of the palisade parenchyma, and vascular bundles towards the galls. Resin ducts became abundant (Figure 4A).

The larval chamber region showed an intense hypertrophy of the palisade parenchyma and the development of nutritive tissue from a meristematic tissue with 2–3 layers through the dedifferentiation of the adjacent parenchyma (Figure 4A,B,G). In the mature stages of development, this portion was composed of 2–3 layers of flattened parenchyma cells towards the surface and the beginning of degeneration of the epidermis, hypodermis, and palisade parenchyma to form the exit channel (Figure 4C–F). Finally, in the senescent stage of development, the exit channel exhibited abundant hyphae. Meristematic cells were found from the hypodermis towards the larval chamber around the exit channel and degenerated nutritive tissue (Figure 4H).

**Figure 5:**
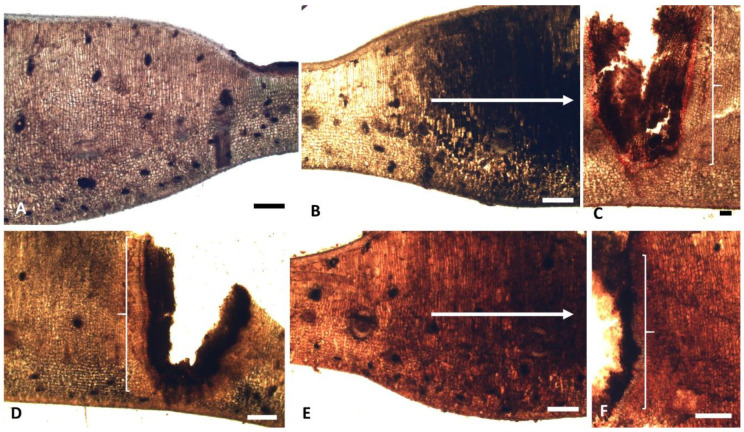
*C. fluminensis*. Galled leaves of male individuals (**A**–**F**). TS exhibiting histochemical analyses. (**A**) Blank section. (**B**) Test with FeCl3 exhibiting phenolic compounds (dark precipitate). Note the abundance of phenolics towards the larval chamber (arrow), notably in the palisade and spongy parenchyma. (**C**) Test with Sudan IV revealing lipids in the adjacent layers of the nutritive tissue (}) but rare in the mesophyll. (**D**) Test with lugol, revealing starch (dark precipitate) in the nutritive tissue and adjacent layers ({). (**E**,**F**) Test with Fehling and Benedict reagents showing reducing sugars (brownish precipitate). Note the abundance in the mesophyll towards the larval chamber (}) (arrow). Bars: (**A**–**F**) = 200 µm.

The histochemical analyses (Figure 5A–F) revealed starch in the nutritive tissue and adjacent layers, and next to ducts (Figure 5D). Lipids were found in the epidermis and adjacent layers of the nutritive tissue but were rare in the mesophyll (Figure 4G and Figure 5C). Phenolics (Figure 5B) and reducing sugars (Figure 5E,F) were abundant in all tissues next to the larval chamber, notably in the palisade and spongy parenchyma, with the latter also being found in the nutritive tissue.

### 2.3. Chemical Analyses

The chemical analyses revealed no differences in the galled and non-galled leaves of both sexes regarding the presence of flavonoids, tannins, steroids and triterpenes, resins, and quinones, whereas saponins and alkaloids were absent. The tests for flavonoids revealed the presence of flavones, flavonols, xanthones, chalcones, aurones, leucoanthocyanidins, and flavanons, whereas anthocyanins, anthocyanidins, flavanonols, and catechins were absent. Higher extractives were found in females (8.44%) than in male leaves (3.91%).

The total phenolics and flavonoids are summarized in Table 1. Significant differences were found between the phenolics of leaves of male individuals, with a higher concentration in galled and adjacent opposite leaves than the non-galled leaves. Furthermore, female individuals produced significantly more phenolics than male ones.

## 3. Discussion

### 3.1. Morphology

There were significant changes in the morphology of the leaves with galls due to the activity of the gall inducing Cecidomyiidae larvae. The observations indicated that the development of several galls may affect the surface of the leaf blade of the galled leaves. The impacts over morpho-physiological traits of leaves caused by Cecidomyiidae galls have also been registered in *C. lanceolata* [15,23]. These studies indicated that variation in parasitic intensity created differences in leaf response, such as increased leaf biomass and a decrease of the specific area under moderate attack.

We did not register the oviposition of the inducers. Nevertheless, the initial stages of gall development were detected in young leaves, which corroborates the beginning of gall differentiation in young tissues [34] since they are more reactive to gall induction than already differentiated tissues [35].

The surface of galls became rough, with thick cuticle and crusts of epicuticular wax, which indicate additional protection to them. The epicuticular wax plays an essential role in water loss regulation since transpiration through cuticle is related to its permeability and the habitat where the plant develops [36,37]. Furthermore, the epicuticular wax may also be waterproof to the environmental humidity, avoiding water penetration into the intercellular spaces and growth of endophytic microorganisms [38].

In the initial stage of gall development, the leaves showed injuries in the epidermis of galls, which indicated the penetration of the gall-inducers. Nevertheless, we did not observe wound tissues in the epidermis of galls which might reflect other penetration sites of the inducers. Several cork-warts developed in both faces, especially the abaxial surface of galls. These structures were also registered in *Clusia* [39]. They are rich in suberin and may have distinct origins [40], arising from stomata [41,42], hairs [43], and from injuries caused by insects [44,45]. Thus, the harm caused by the penetration possibly triggers the formation of such structures. Furthermore, cork-warts were more abundant in the epidermis of galls than the remaining non-galled area, probably related to the hypertrophy of the leaf blade.

The galled leaves showed hypertrophy of the mesophyll, with new layers of parenchyma cells from the hypodermis and the palisade parenchyma. The presence of highly vacuolized cells indicates water and metabolites reservoir for the development of the galls, in addition to mechanical protection. The homogenization of the parenchyma and cell hypertrophy is commonly described in leaf gall formation as transformation patterns into nutritive and protective tissues [14,35,46,47]. An interesting feature is the formation of meristematic tissue surrounding the larval chamber, representing the continuous source of cells to the nutritive tissue and adjacent region. In the late stages of development, this meristematic reservoir is responsible for forming sclerified and suberin rich cells that isolate the remaining living tissues. Then, a coordinated programmed cell death across the mesophyll takes place to develop the upcoming exit channel. Such organized development since the early stages of development involves underlying genetic control mechanisms, hormone balance, and chemical signaling under the influence of the Cecidomyiidae larva. Several studies indicated that gall-inducing insects use chemical signaling, although the proper mechanisms remain poorly understood. In leaf galls of *Vitis riparia* Michx. (Vitaceae), the inducer (phylloxera) redirects reservoir cells from the vascular cambium to develop new meristematic tissue. It drives leaf development towards flowering pathways, such as carpel formation. The inducer uses genetic machinery in the host plant for its benefit [48]. We may also observe the inverse, in which flower organogenesis is driven towards leaf development pathways in different floral galls in *Byrsonima sericea* DC. (Malpighiaceae) [15,16].

Idioblasts with drusa are abundant in the subepidermal layers, scattered across the mesophyll, vascular tissues, and the exit channel. Shorthouse [49] and Kraus [17] suggested that crystals and sclerenchyma cells increase mechanical protection to the gall maker. We observed several resin channels in the mesophyll and petiole of galled and non-galled leaves of both sexes. A remarkable feature of Clusiaceae is the presence of laticifers in all tissues that produce lipids, essential oils, alkaloids, and resin [10]. We registered the development of several new laticifers towards the gall tissues, which indicated their relation to the gall-inducer system’s nutrition.

The vascular system of petioles of galled leaves was not significantly different from the non-galled ones. However, we observed the hypertrophy and formation of new collateral bundles towards inner gall tissues, the larval chamber, and adjacent tissues. New vascular tissues are related to increased nutrients required by the gall system [49], especially new sieve tubes, which often develop below the inner gall surface, supplying and enveloping the nutritive tissue [47]. Thus, the maintenance of the vascular tissues indicates that the necessary further resources and nutrients were obtained from nutrients metabolized and stored in the galled leaves.

The exit channels arose from coordinated programmed cell death of parenchyma cells and hypodermis in the senescent stages and remained isolated from the remaining tissues. Hyphae became abundant in the exit channel, which indicates the lowering of chemical defenses after the departure of the inducer. We did not register the degeneration of the remaining gall tissues, besides those isolated in the exit channels, which indicates that the senescent features were local, and the remaining tissues of the leaf blade remain physiologically active.

One of the most interesting insights is that we did not register galls in female individuals. According to Watson [25], male individuals tend to be more susceptible to herbivory than female ones due to sex differences in phenology, spatial and temporal distributions, the composition of defense and nutritive metabolites, growth patterns, and the allocation of resources. The investment for reproduction might change the nutritional and defense levels and growth rates, even in similar habitats. Boecklen et al. [50] observed gall-inducing wasps with a preference for leaves of male individuals in *Salix lasiolepsis* Benth. (Salicaceae).

The plant host–gall inducer interactions and the influence of sex on the response to galls have been registered in different environments. To test the “sex-biased herbivory hypothesis,” which states that male individuals could support a greater abundance of galls than female ones, a study in *Baccharis concinna* Barroso (Asteraceae) revealed no significant differences between male and female plants regarding the number of galls [51]. According to the authors, other variables besides host sex may influence induction patterns by galling herbivores. In *Baccharis dracunculifolia* DC, the sex of host plants was not related to tannin gradients and abundance and mortality rate of galls, which indicated that the abundance of galls was probably influenced by other secondary metabolites or mechanical defenses [52]. Also, Ribeiro-Mendes et al. [53] suggested that host plant sex was not related to the abundance of galls. *B. dracunculifolia* exhibited no significant differences in growth rates or nutritional status between male and female plants. Other species, *Baccharis pseudomyriocephala* Teodoro, showed no influence of sex regarding abundance and richness of gall-inducing insects [54].

The individuals of both sexes of *C. fluminensis* exhibited similar leaf anatomy and phenology, corroborating the pattern described in the literature with no significant gender differences [55]. Thus, the factors responsible for preferential development in male individuals were probably related to sex-biased metabolic variation and chemical composition of leaves.

Finally, the absence of galls in female individuals may reflect on their fitness and other ecological aspects on the community-level due to their potential impacts on morpho-physiological traits, such as photosynthesis and available resources to reproduction. *Clusia* represents an important floristic element, and several species act as nurse plants, especially *C. hilariana* and *C. fluminensis* at “restinga” habitats [8,10,56]. Possible gender differences in ecophysiological performance and their effects on their role as nurse plants were evaluated in the dioecious *C. hilariana*. Similar ecophysiological traits and high species diversity were found between male and female individuals [56]. Nevertheless, further studies are necessary to confirm how the sex-biased gall development described herein may reflect on such ecological aspects of *C. fluminensis.*

### 3.2. Histochemical Analyses

Our results indicated that the new gall tissues presented high phenolics levels. Gall inducers influence and benefit from the increase of phenolics, especially tannins, in galled tissues [27,57].

We observed the accumulation of lipids in galled tissues and higher starch and sugars levels, especially in the palisade towards the larval chamber. Lipids are generally found in Cinipidae galls, whereas carbohydrates are observed in Cecidomyiidae galls [58]. Similar exceptions to this proposed pattern were also found in *C. lanceolata* [14] and *Lantana camara* L. (Verbenaceae) [59]. The storage of lipids next to the nutritive tissue in *C. fluminensis* indicated a nutritious gradient for the larva. Nevertheless, they are also related to the maintenance of the gall since lipids represent reservoir molecules with high energy levels and precursors of essential components of plant metabolism [60].

Cecidomyiidae galls usually exhibit nutritive tissues free of starch and present in peripherical tissues [58]. However, our analyses revealed starch in the nutritive tissue. Such histochemical gradients represent a response to the parasites and may occur a temporal and spatial variation in the chemical composition of galled tissues. Furthermore, the quality of galled tissues may be associated with the age of leaves. Oliveira and Isaias [61] observed the variation of the histochemical gradient and the development of galls in *Aspidosperma australe* Müll.Arg. (Apocynaceae). They suggested that such differences are a response to the activity of the inducer. Another study with different Cecidomyiidae galls in *Copaifera langsdorffii* Desf. (Fabaceae) registered similar results, in which the histochemical gradient in galled tissues was related to the inducers [62]. The higher nutritional value of neo-developed tissues, especially those adjacent to the larval chamber, arises from synthesizing structural and enzymatic proteins and concomitant proteolysis, which increases soluble amino acids and nitrogen [47,63,64]. These products may also come directly from the host plant through the vascular system, through the several new vascular bundles and laticifers towards the gall described herein.

Cecidomyiidae galls in *C. langsdorffii* showed lower nitrogen and starch and increased sugars and polysaccharides, which confirms their role as drainages of photo-assimilated compounds. According to the nutrition hypothesis related to the adaptive nature of galls [65,66], the inducers present advantages over other herbivores since the galled tissues present more nitrogen than the non-galled ones. Hartley [27] detected that the minority of species with galls exhibited favorable chemical composition to the herbivores. Thus, our results confirmed the histochemical pattern for Cecidomyiidae galls, except for lipids and starch storage, as recorded in *C. lanceolata* [14]. These results key exceptions suggest that lipids and starch storage are related to host plant biochemical pathways, not to Cecidomyiidae inducing factors. Nevertheless, more studies with other host plants are necessary to understand this pattern properly.

### 3.3. Chemical Composition

Our analyses revealed no differences regarding the metabolite classes, especially flavonoids, between the analyzed leaves of *C. fluminensis*. We found triterpenes but no alkaloids and saponins. Such results corroborate the chemical pattern of Clusiaceae. Several species exhibit secretory structures with lipids, terpenes, alkaloids, tannins, and resin [10]. Species of *Garcinia* exhibit laticifers with triterpenes, flavonoids, tannins [67], and saponins and xanthones [32]. We found a higher amount of ethanol extractives in female than male leaves, indicating the higher production of metabolites.

We detected significant differences between total phenolics and flavonoids from leaves of male individuals, with an increase in galled and adjacent leaves compared to those non-galled, which indicates the influence of the Cecidomyiidae over the phenolics synthesis in galled and even adjacent leaves. Additionally, female individuals produced a significantly higher amount of such compounds than male ones, which suggests a sex-biased metabolic variation. Gender differences may explain sex influence over herbivory in dioecious species [25].

Although the role of phenolics in galls is not fully understood, these compounds, such as tannins, are related to defense inhibition [68]. Herbivorous insects also need to manipulate and reduce plant defensive responses to enhance their fitness [27,57,65,66]. Gall-inducing insects also need to escape mortality imposed by other gall inhabitants and outer natural enemies [69]. Since they can manipulate plant development, some of their metabolic changes in gall tissues should also confer protection against these natural enemies [69,70]. Parasitoids were absent in all analyzed galls. These insights indicate that the Cecidomyiidae larva may cope with the increase of phenolics, notably flavonoids, which inhibit the development of other organisms. Nevertheless, they remained sensitive to an even higher dose of phenolics and the synthesis of specific flavonoids in female individuals, which may explain the absence of galls.

Nevertheless, the gall-makers benefit from the increase of phenolics and tannins in galls [57] and influence the chemical composition of galled tissues, especially over phenolic compounds. Such metabolites protect the inducers from parasitoids, fungi, and other microorganisms [71]. They can act in pollination, UV protection, and hormone regulation [17,66]. They also present indirect benefits for the larva by increasing auxin levels, which contributes to cell growth and redifferentiation in galls [72]. Finally, they may inhibit the hypersensitive reaction, mitigating damages from free radical species in oviposition sites [73]. Similar results were found in *Aspidosperma spruceanum* Benth. ex Müll.Arg., in which the inducers, along with abiotic factors, influenced total phenolics in the infested leaves [74].

Additionally, such variation in flavonoids composition indicated changes in metabolic pathways. A similar variation in flavonoids composition was found in leaf galls induced by Hymenoptera in *Salix* species, in which only traces of flavonols and flavones were found in the galls. The reduction of such compounds was related to the blockade of their pathways [75]. The Hessian fly *Mayetiola destructor* Say, 1817 (Cecidomyiidae) counteracts plant defenses by using phenylpropanoid pathways for its benefits in wheat. The study revealed the downregulation of most protease inhibitor genes and others related to the hostplants’ phenylpropanoid pathway, resulting in lower chalcone and flavonoids isoflavonoids and lignin at the insect feeding site [68]. These metabolic changes benefit the development of larva since several of these phenylpropanoids are toxic to insects [76].

Flavonoids may also act as chemical barriers, specifically as a feeding inhibitor of the larva [77]. Another study in two species of *Aspidosperma* registered the increase of phenolics, including flavonoids [78]. The variation in such compounds in both species was related to defense strategies against the gall-inducer insects. Unlike *A. australe*, which was attacked by such parasites, *A. cylindrocarpon* exhibited a higher number of flavonoids and sclereids and the absence of galls. The study suggested that phenolics, especially flavonoids, alongside the anatomical features prevented larva feeding and, thus, the development of gall tissues.

The absence of galls in female individuals of *C. fluminensis* is probably related to the higher amount of phenolics and flavonoids, which provides higher chemical protection against Cecidomyiidae larva than male individuals. Furthermore, the rise in flavonoids in galled leaves is a plant response to the inducer, as an attempt to limit the larva activity and lower their impacts over galled leaf tissues.

Most recent studies focused on insect strategies to deal with plant chemicals, including excretion, sequestration, and phytotoxins degradation. However, the direct modulation of plant metabolism by insects to cope with plant defenses at source was seldom described [47]. Thus, the mechanisms that manipulate host plant defense levels remain poorly understood in any insect-gall system. We believe that it represents a novel frontier in further studies regarding gall development, and leaf galls in *C. fluminensis* represent an interesting case study.

One of the critical features in gall development is the early gall differentiation in young tissues [34] as they are more reactive to gall induction than already differentiated tissues [35]. Nevertheless, the proper ways that gall inducers regulate such intricate plant organogenesis changes must be adequately understood in both vegetative and reproductive organs. However, if we consider the advances in plant development regulation pathways, we must consider specific factors and balance of phytohormones. Saliva or mechanical injuries could promote such changes. Several studies assessed whether insect effectors present in oral saliva might play a role in host plant transformation [79,80,81] and the subsequent consequences on insect performance. Some of these effectors have already been described. However, their specific chains of interaction in the host plants often remain unclear.

Additionally, several studies indicated changes in plant hormone balance over the past decades, in different gall inducers, such as auxin and gibberellin; abscisic acid in *Phragmites australis* (Cav.) Trin. ex Steud. (Poaceae) induced by Diptera [82]; exogenous phytohormones [83] and also auxin: cytokinin balance in floral galls caused by fungi in *B. sericea* [16]. Nevertheless, the mechanisms by which the inducers, mostly insects, promote gall induction and growth by manipulating hormones and, consequently, gene expression regulation are mostly unresolved. Insects might be the source of hormones instead of driving hormone balance and signaling, which allows them to use plant machinery effectively [47].

Finally, studies regarding gall development focused on the mechanisms that regulate gene expression and, consequently, metabolic and morphological changes may unveil the target spots of host plant metabolism. Nevertheless, we stress that histochemical and morphological studies are still necessary and one of the best ways to understand development patterns, notably in such complex interactions as gall inducer-host plants.

## 4. Materials and Methods

### 4.1. Study Site and Sampling

The Brazilian *restinga* covers nearly 5000 km along the coast, representing 79% of the country’s coastal land. This biome presents variable physiognomy, ranging from herbs and shrubs to forest formations, with discontinuous, open sand areas [1,2,4]. Field observations and collection of material were conducted in the Barra de Maricá Environmental Protection Area (22°52′–22°54′ S and 42°48′–42°54′ W, total area 8.3 km^2^ at sea level) in Rio de Janeiro, Brazil, monthly from January 2011 to June 2013. For the identification and follow-up of leaf galls since the first stages to the senescent stages of development (March–June), vegetative branches in five individuals of each sex of *C. fluminensis* were marked while still in the budding stages. The collected branches had galled and non-galled leaves at all stages of development, with at least 30 leaves for further morphological and chemical analyses. To assess the possible effects of galls in adjacent organs, the adjacent opposite leaves to the galled ones were also evaluated. The exsiccates of male and female individuals were deposited in the herbaria of the Museu Nacional (R), under vouchers R217428 and R217429, and Instituto de Biologia (RFA), under vouchers RFA 39552 and RFA39553, at Universidade Federal do Rio de Janeiro.

### 4.2. Morphology and Anatomy

We measured leaf galls in different (initial, mature, and senescent) development stages with a microcaliper and fixed in 4% paraformaldehyde and 2.5% glutaraldehyde in 50 mM pH 7.2 phosphate-buffered saline [84] to understand their ontogenesis. Each stage was determined in terms of the size and hypertrophy of the leaf blade for the initial and mature stages, and the exit channel and absence of the inducer for the senescent stage. For scanning electron microscopy (SEM), the samples were fixed in ethanol series, dried to the critical point of CO_2_ at 73 atm and 35 °C using Leica EM CPD030 equipment (Leica Microsystems, Inc., Buffalo Grove, IL, USA), and coated in gold [85] with a Denton Vacuum—Desk IV (Dentom Vacuum, LLC, Moorestown, NJ, USA). The observations and documentation of material were conducted with scanning electron microscope JEOL JSM—5310 (JEOL USA, Inc., Peabody, MA, USA). For light microscopy, the samples fixed in diverse development stages were dehydrated in alcohol series and embedded in Paraplast^®^ (Sigma-Aldrich, Co., St. Louis, MO, USA) [84]. Serial sections 9–14 µm thick, obtained with a rotation microtome, were stained with 1% astrablue and 1% safranin (9:1, *v*/*v*) [86]. For the histochemical analyses, samples were sectioned by hand with a Ranvier microtome. The sections were submitted to the following tests: (i) starch detection with Lugol reagent [87]; (ii) detection of phenolic substances with iron chloride solution [88]; (iii) detection of lipids with Sudan IV [88]; (iv) detection of sugars with Fehling and Benedict reagents; (v) investigation into the chemical nature of the crystals with 10% acetic acid and 10% nitric acid [89]. For the leaf epidermis study, fragments from galls and intercostal region of non-galled leaves of either sex were dissociated with 30% hydrogen peroxide and acetic acid (1:1) [84]. Then, they were stained with 1% safranin. We mounted the slides of freehand sections in 50% glycerine and the embedded ones in Entellan^®^ (Merck KGaA, Darmstadt, Germany). The follow-up study and obtaining images were performed with stereomicroscope Taimim TM 99000777 (Taimin, João Pessoa, Brazil) with Motican 2300 photographic equipment (Motic Incorporation Ltd., Causeway Bay, Hong Kong) and optical microscope Carl Zeiss Axio Scope A1 with a capturing system composed of AxioCamERc 5s and Zen Lite image software (Carl Zeiss MicroImaging GmbH, Gottingen, Germany). The classification of shapes was based on Radford et al. [90] and Wilkinson [91].

### 4.3. Chemical Analyses

For chemical prospecting and evaluation of the possible effects of galls regarding the secondary metabolites’ classes, plant material was obtained from five specimens of each sex and dried at 40 °C. Then, 40 g of dried material of each whole organ (non-galled leaves and leaves with galls in mature stage of development) were macerated in 100 mL of 70% ethanol for five days at 25 °C. The filtered sample was dried with a rotary evaporator (60 °C; 100 rpm). We used the protocols established by Matos and the Brazilian Pharmacopeia [92,93] to detect flavonoids, alkaloids, sterols, and free triterpenes (Lieberman-Burchard reaction), saponins, tannins, and resins. For quinones detection, we performed the Bornträeger reaction [94]. We used the foam index (>1 cm) in test tube after agitation to detect saponins. For tannins detection, we added the reagents FeCl3 10% and Pb(C_2_H_3_O_2_)_2_ 10% into each test tube. For flavonoids detection, we added 3 mL of each extract into three numbered test tubes, following pH changes with HCl 5% or NaOH 3% (3.0, 8.5 and 11.0, respectively). The evaluation of each type of flavonoids was based on the color changes according to the pH range. For the positive controls, we used extracts of *Achyrocline satureioides* (flavonoids), *Geissospermum leave* (alkaloids), *Frangula purshiana* (quinones), *Psidium guajava* (tannins), *Ziziphus joazeiro* (saponins and triterpenes), *Nerium oleander* (sterols), and *Clusia lanceolata* (resins). Blank extracts were used for comparative analysis.

### 4.4. Thin-Layer Chromatography (TLC)

Extracts of non-galled leaves of both sexes, leaves with mature galls, and adjacent opposite leaves to galled leaves of male individuals were analyzed. For partitioning and identification of chemical compounds, we used TLC plates and aluminum sheets (AL TLC 20 × 20 cm Silica gel 60 F254 Merck) with eluent solutions of butanol, acetic acid, and water (10:2:5) [92,93,95]. For the detection of flavonoids, we used AlCl_3_ in UV (365 nm).

### 4.5. Determination of Phenolic Compounds and Flavonoids

To evaluate the total amount of phenolics in leaves (non-galled leaves; leaves with galls in mature stage of development, and adjacent opposite leaves to the galled leaves), we used a spectrophotometer BEL SP200 UV (Bel Photonics, Osasco, Brazil) with Folin-Ciocalteau reagent [96]. The phenolic compounds were obtained from 1 g of dry material with successive extractions with 15 mL of ethanol 70% at 60 °C for 15 min until the 50 mL volume. Aliquots of 20, 40, 80 µL of each extract were mixed to 2.5 mL of 10% Folin-Ciocalteau reagent and 2 mL of Na_2_CO_3_ solution for 2 h in the dark. The flavonoids were obtained from the TLC analyses [92,95], which were performed with aliquots of 80 µL of each extract in TLC plates. They were then removed and dissolved in 1 mL of 70% ethanol, and aliquots of 500 µL were submitted to the previous protocol of Folin–Ciocalteau reagent. The absorbances were measured in a spectrophotometer (760 nm) [97]. The assays were performed in triplicates. The calibration curve was obtained with aliquots of 50, 100, 150, 200, 250, 300, 350, 400, 450 e 500 μg/mL of Gallic acid in distilled water. The total amount of phenolics and flavonoids were determined by the equation of line obtained in the calibration curve in Excel^®^.

### 4.6. Statistical Analysis

To evaluate the effects of galls on the chemical composition of leaves, especially the total phenolics and flavonoids, we performed ANOVA and post-hoc Tukey’s test between the different types of leaves in male and female individuals. Statistical analyses were performed using Statistica 8.0 (Statsoft, Inc., Tulsa, OK, USA).

## 5. Conclusions

This paper describes how galls affect the development and metabolism of phenolic compounds in a dioecious plant from Brazilian *restinga*. We stress that the results described here are a step towards explaining different kinds of plant–parasite interaction and a significant contribution to bridging the gap in the literature on the study of galls with diverging patterns in dioecious plants from the neotropical region. We describe a set of changes in the morphology and chemical composition of galled leaves of *C. fluminensis*. Our results indicated an interesting exception for the general pattern of storage of lipids and starch in Cecidomyiidae galls, and sex-biased metabolic variation, which inhibits gall development in female individuals. In the future, we hope to contribute further studies on parasite–host plant relationships and their possible effects on the metabolism of different dioecious plant species.

## Figures and Tables

**Table 1:** Total contents of phenolics and flavonoids from samples of different types of leaves in both sexes of *C. fluminensis.* Numbers are the mean value of the metabolite class (mg/g; dry mass) and the standard deviation. Means with different letters within a column are significantly different at the 0.05 probability level (ANOVA and post-hoc Tukey test).

Species (Type of Individual—Type of Leaves)	Phenolics (mg/g)	Flavonoids (mg/g)	Flavonoids (%)
Male individuals—non-galled leaves	10.15 ± 0.31 a	6.75 ± 0.04 a	66
Male individuals—galled leaves	14.74 ± 0.42 b	7.19 ± 0.04 b	49
Male individuals—adjacent leaves	12.90 ± 0.42 c	6.88 ± 0.04 a	53
Female individuals	16.29 ± 0.66 d	7.54 ± 0.09 c	46

## Data Availability

Data sharing not applicable.
